# Piezoelectric Micromachined Ultrasound Transducer (PMUT) Arrays for Integrated Sensing, Actuation and Imaging

**DOI:** 10.3390/s150408020

**Published:** 2015-04-03

**Authors:** Yongqiang Qiu, James V. Gigliotti, Margeaux Wallace, Flavio Griggio, Christine E. M. Demore, Sandy Cochran, Susan Trolier-McKinstry

**Affiliations:** 1 Institute for Medical Science and Technology, University of Dundee, Dundee DD2 1FD, UK; E-Mails: y.qiu@dundee.ac.uk (Y.Q.); c.demore@dundee.ac.uk (C.E.M.D.); 2 Materials Research Institute, The Pennsylvania State University, University Park, PA 16802, USA; E-Mails: jamey.gigliotti@gmail.com (J.V.G.); margeaux.wallace@gmail.com (M.W.); flavio.griggio@gmail.com (F.M.); stmckinstry@psu.edu (S.T.-M.); 3 Materials Science and Engineering, Georgia Institute of Technology, Atlanta, GA 30332-0245, USA; 4 Intel Corporation, Hillsboro, OR 97124, USA

**Keywords:** ultrasound, piezoelectric thin films, micromachined ultrasound transducers, arrays, integrated transducers

## Abstract

Many applications of ultrasound for sensing, actuation and imaging require miniaturized and low power transducers and transducer arrays integrated with electronic systems. Piezoelectric micromachined ultrasound transducers (PMUTs), diaphragm-like thin film flexural transducers typically formed on silicon substrates, are a potential solution for integrated transducer arrays. This paper presents an overview of the current development status of PMUTs and a discussion of their suitability for miniaturized and integrated devices. The thin film piezoelectric materials required to functionalize these devices are discussed, followed by the microfabrication techniques used to create PMUT elements and the constraints the fabrication imposes on device design. Approaches for electrical interconnection and integration with on-chip electronics are discussed. Electrical and acoustic measurements from fabricated PMUT arrays with up to 320 diaphragm elements are presented. The PMUTs are shown to be broadband devices with an operating frequency which is tunable by tailoring the lateral dimensions of the flexural membrane or the thicknesses of the constituent layers. Finally, the outlook for future development of PMUT technology and the potential applications made feasible by integrated PMUT devices are discussed.

## 1. Introduction

**Figure 1:**
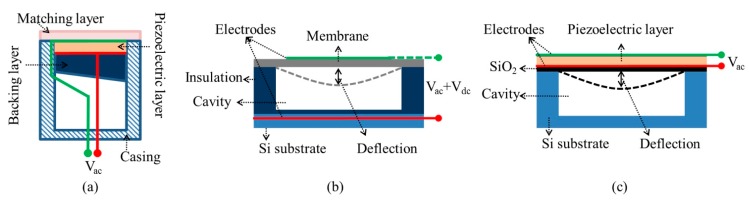
Typical cross-sectional structures of (**a**) piezoelectric ultrasonic transducers; (**b**) CMUTs and (**c**) *d_31_*‑mode PMUTs.

In the last several decades, ultrasound has found ever-expanding industrial and biomedical applications, such as non-destructive evaluation (NDE) [1,2], ultrasonic actuation [3], medical imaging [4,5], therapeutic ultrasound [6], and particle and cell manipulation [7,8], at frequencies from tens of kilohertz to hundreds of megahertz. Ultrasound can be excited by many different methods, including the piezoelectric effect, magnetostriction, and the photoacoustic effect [9,10]. Of these, the piezoelectric effect is the most common. A typical structure of a conventional piezoelectric ultrasonic transducer is shown in Figure 1a. It usually has a layer of piezoelectric material sandwiched by thin high conductivity electrode layers, of e.g., Au or Pt, often with an underlying adhesion layer e.g., of Cr or Ti, and connected with electrical wires.

For this kind of transducer, the longitudinal vibration mode (*d_33_*-mode) of the piezoelectric material is utilized. Thus the anti-resonant frequency of the transducer is related to the thickness of the piezoelectric layer and the longitudinal velocity of sound in the poling direction of the piezoelectric material [11]. This direct dependence of the resonant frequency on the layer thickness therefore limits the transducer geometry and structure for specific applications. For example, the resonant frequency of a thin film of lead zirconate titanate (PZT) in the *d_33_*‑mode is at least several hundreds of megahertz. This ultrahigh frequency may benefit ultrasound microbeam cell manipulation [12]. However, the high attenuation associated with this frequency range limits the penetration depth of ultrasound in tissue and thus it is very rarely used in ultrasound imaging applications. In addition, acoustic impedance (*Z_ac_*) mismatching between the piezoelectric layer (*Z_ac_* > 30 MRayl for conventional PZT) and the load medium, e.g., air (~400 Rayl), water (~1.5 MRayl) or soft tissue (~1.6 MRayl) [13], can limit the energy transmission and reduce the bandwidth, even with matching layers placed on the front face of the piezoelectric material. This also ignores the manufacturing difficulty and complexity of matching layers with precise thickness for high frequency ultrasound transducers [10,14,15]. Furthermore, the fabrication difficulty of conventional piezoelectric transducer structures increases dramatically for 1-D linear and 2-D matrix ultrasound arrays for beam steering and 3-D volumetric imaging [5,16], and in miniaturized devices with high element density to be fitted into small, constrained spaces, e.g., in intravascular ultrasound (IVUS) and intracardiac echocardiography (ICE) catheters [17,18,19].

Many of these problems can potentially be overcome by micromachined ultrasound transducers (MUTs). The MUT family includes capacitive micromachined ultrasonic transducers (CMUTs) based on the flexural vibrations caused by a field-induced electrostatic attraction between suspended membrane and the substrate (Figure 1b) [20,21,22,23,24,25], and piezoelectric micromachined ultrasonic transducers (PMUTs) based on flexural vibrations caused by *d_31_*‑ [26,27,28,29,30,31,32,33,34,35,36,37,38] or *d_33_*‑mode [39,40,41] excitation of a piezoelectric membrane (Figure 1c).

A CMUT element is, in essence, a miniaturized capacitor that consists of a thin metallized suspended membrane, e.g., silicon nitride (Si_x_N_y_), over a cavity with a rigid metallized substrate, e.g., silicon (Si), as shown in Figure 1b. When a DC voltage is applied between the two electrodes, the membrane is deflected, being attracted toward the substrate by electrostatic forces. The mechanical restoring force caused by the stiffness of the membrane resists the attraction [25]. Consequently, ultrasound can be generated from the oscillations of the membrane with an AC voltage input.

In contrast, the deflection of the membrane in the PMUT is caused by the lateral strain generated from the piezoelectric effect of the membrane, which must thus include at least one piezoelectric layer, typically a thin PbZr_1-x_Ti_x_O_3_, PZT, film [30,42], as well as a passive elastic layer. In this case, the resonant frequency of the PMUT does not directly depend on the thickness of the piezoelectric layer. Instead, the flexural mode resonant frequencies are closely related to the shape, dimensions, boundary conditions, intrinsic stress and mechanical stiffness of membranes, as they are also in CMUTs.

Intrinsic stress in the membrane generated during fabrication can affect the resonant frequency dramatically. In the case of an edge-clamped circular diaphragm with low intrinsic stress, the membrane behaves as a plate with the resonant frequencies, *f*, given as [43,44,45]:

(1) $$f=\frac{\text{α}}{2\pi r^{2}}\sqrt{\frac{D_{E}}{\rho h}}$$

(2) $$D_{E}=\frac{Eh^{3}}{12(1-{\text{υ}}^{2})}$$

where α is the resonance mode constant, *r* is the radius of the diaphragm, *D_E_* is the flexural rigidity, ρ is the effective density of the diaphragm, *h* is the diaphragm thickness, *E* is the effective Young’s modulus, and υ is Poisson’s ratio. With high intrinsic stress, *T*, the stress can dominate over the flexural rigidity, hence the membrane behaves as a membrane with no bending stiffness with the resonant frequencies given as [43,44,45]:

(3) $$f=\frac{\text{α}}{2\pi r}\sqrt{\frac{T}{\text{ρ}h}}$$

Therefore, the resonant frequency of the MUTs can be controlled with proper selection of the radius of the diaphragm with tailored effective mechanical stiffness, leading to better design flexibility regardless of any thickness restrictions of the piezoelectric layer. Since the bandwidth may be narrow in some designs, it is also possible to arrange a set of MUTs with different diaphragm sizes and frequencies to broaden the bandwidth and improve upon the sensitivity of a single device [34]. A soft membrane with tuneable *Z_ac_* leads to better impedance matching with the load media, thus theoretically improving the energy transmission and bandwidth [24]. Through wafer-scale microfabrication [46], miniaturized transducer arrays with high element density can be fabricated to meet the functional and geometrical requirements of new applications [21,35]. Microfabrication techniques also give the possibility to integrate MUTs with circuitry and front-end electronics in the same chip [21,24].

CMUTs have been demonstrated to produce high bandwidths (up to 175%) and electromechanical coupling coefficients (~0.85) and output acoustic pressure exceeding conventional transducers [20,23,24,47]. However, in practice, the high performance can only be achieved when a large DC bias near the so-called collapse voltage is applied, which increases the risk of failure of the device. This can, to some extent, limit performance in biomedical applications. Although new driving methods have been demonstrated [48], the problem remains. Moreover, different CMUT designs may be required for transmission and reception because of different cavity height requirements; thus, different transmission and reception arrays may be required for imaging applications [30].

Unlike CMUTs, PMUTs do not require a large voltage bias and have fewer geometric and design constraints, facilitating integration with low voltage electronics. PMUTs also offer several other advantages over CMUTs, largely because of their higher capacitance and lower electrical impedance. These increase the transducer sensitivity by decreasing the effects of parasitic capacitance while enabling use of low voltage electronics [30,32,49,50]. Large output signals, low loss and high signal-to-noise ratios (SNR) can be further achieved with the implementation of piezoelectric thin films [37,42,51]. Although printed thick film piezoelectric materials can lead to low cost devices, it is usually with a loss of performance. Furthermore, challenges remain for integrated electronics and fine-scale interconnects for miniaturized devices [52].

Despite the promise of PMUTs, the high sensitivity of the resonant frequency to the residual stress of the membrane may cause difficulties during the design process [34,49,53,54,55]. Also, it is essential to locate the neutral axis outside the piezoelectric layer to maximize the net polarization change in the receive mode [29]. Therefore, the metallized piezoelectric layer is usually deposited on passive elastic layers such as Si, Si_x_N_y_ or SiO_2_. The optimal layer thicknesses and configuration identified from modelling can ensure maximum sensitivity of the piezoelectric layer and allow for better tuning of the mechanical stiffness of the membrane.

To date, several research groups have successfully developed PMUTs with different fabrication approaches and achieved promising results. Most PMUTs are fabricated using similar techniques, with altered processes mainly dependent on the chosen substrates and thin film piezoelectric materials. This paper presents an overview of recent advances towards the development of miniaturized and integrated arrays with PMUT technology, including a review of a specific new process. In the following sections, the requirements of the piezoelectric materials and variety of possible fabrication methods for PMUT elements and arrays are discussed.

## 2. Piezoelectric Materials for PMUTs

As is the case for conventional ultrasound transducers, the piezoelectric material most widely used in PMUTs is PZT, a ferroelectric material first formulated in the 1950s by Jaffe [56]. Although other materials (e.g., PVDF [57,58], AlN [59,60] and ZnO [27,61,62,63,64]) have been relatively commonly reported in PMUTs, especially in early devices, these usually have a weaker piezoelectric response than ferroelectric oxide films (including PZNT [34,65]). PZT-based compositions are therefore better for low voltage actuation and high sensitivity sensing [42,66].

PZT thin films usually have a thickness between a few nanometers and ~3 µm. In order to deposit material with good ferroelectric properties on a silicon substrate, buffer layers are required to prevent lead diffusion and oxidation reactions [66]. As bottom electrodes are needed for most applications, electrodes such as platinum [35] and SrRuO_3_ [67] may act as one of the buffer layers. A number of coating methods have been adapted for the deposition of PZT thin films, including physical methods such as ion beam sputtering, RF magnetron sputtering, and pulsed-laser deposition (PLD), and chemical methods such as sol-gel deposition, metal-organic-decomposition (MOD) and metal-organic-chemical vapor deposition (MOCVD) [66]. Both sputtering [36,68] and sol-gel deposition [26,28,32,37,69,70,71] methods have often been used to prepare PZT thin films with large piezoelectric coefficients and high remanent polarization for use in PMUTs.

**Figure 2:**
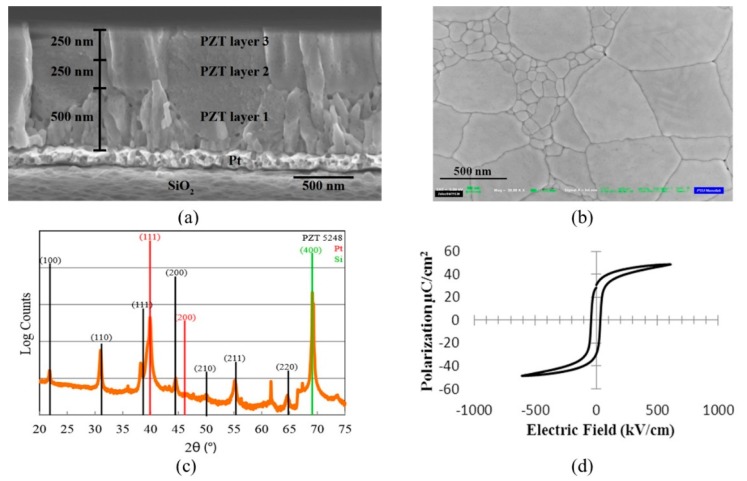
FESEM images of (**a**) cross-sectional and (**b**) top surface of PZT thin film; (**c**) XRD pattern of the PZT film with the absence of PbO (2θ = 29.09°) and pyrochlore/fluorite (2θ = 29.55°); (**d**) hysteresis loop of the sputtered PZT thin film.

As an example, Figure 2 shows a 1 µm thick PZT film sputtered on a Pt/Ti/SiO_2_/Si wafer with an RF sputtering system (CMS-18, Kurt J. Lesker Co., Jefferson Hills, PA, USA). The results of X‑ray diffraction (XRD) and the hysteresis loop of the PZT thin film suggest a high quality was achieved.

The effective piezoelectric response of PZT thin films is usually very different from that of bulk PZT, particularly because of the in-plane clamping of the film by the substrate, as well as from the residual stress in the film [42]. By releasing the substrate beneath the thin film PZT, the mechanical constraints near the bottom interface of the PZT are reduced and the piezoelectric domain walls can more freely respond to the electric field and contribute more to the dielectric and piezoelectric properties, leading to better performance of the PMUTs [36,72]. Partially unclamping diaphragm edges [32,60,73], optimizing electrode configurations [55,74,75] and even adding DC bias [33,76,77] have also been used to increase the coupling coefficients and acoustic output of PMUTs.

## 3. Fabrication of PMUT Element

### 3.1. Diaphragm Defined with Sacrificial Layer Releasing

**Figure 3:**
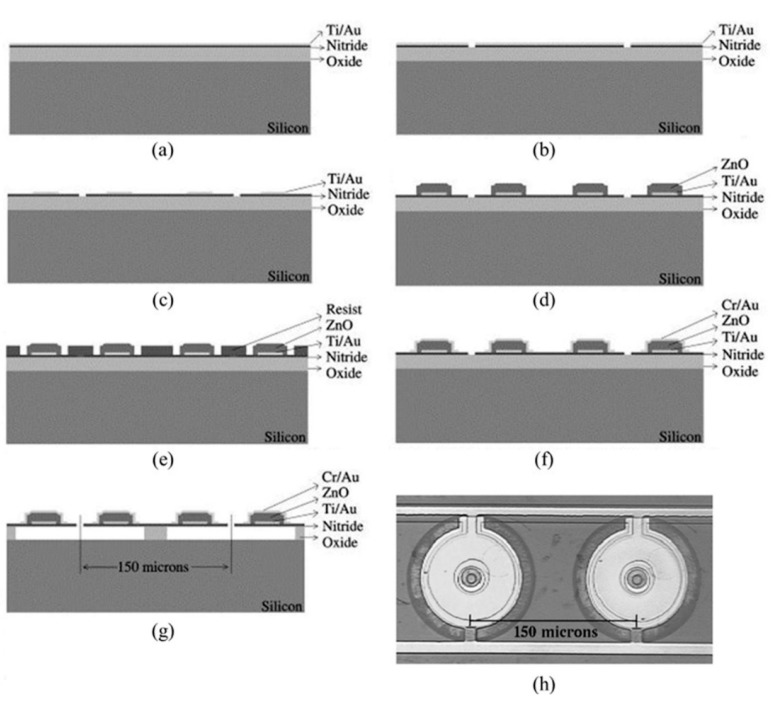
(**a**–**g**) Typical sacrificial layer release process flow; and (**h**) top view of two fabricated adjacent PMUT elements. © 1998 AIP Publishing LLC. Reprinted with permission from [27].

A sacrificial layer release process has been used frequently for microfabrication of both CMUTs [20,22,24,25] and PMUTs [27,62,78]. This usually involves initial sacrificial layer preparation on the substrate. After completing fabrication and patterning of all layers of the diaphragm, it is then released by etching the sacrificial layer through a small opening to form a cavity below the diaphragm. Perçin *et al*. demonstrated the fabrication of ZnO-based PMUT arrays with a low temperature oxide (LTO) as the sacrificial layer [27]. The process is shown in Figure 3.

First, a layer of 8% phosphorus-doped densified LTO was prepared on a Si substrate, followed by a thin layer of silicon nitride (Si_3_N_4_) deposited by low-pressure chemical vapor deposition (LPCVD) and a bottom Ti/Au electrode layer deposited by e-beam evaporation (Figure 3a). Ø8 µm access holes for releasing the final diaphragms were then defined by wet etching of the electrode layer and plasma etching of Si_3_N_4_ (Figure 3b). After patterning the bottom electrode layer (Figure 3c), a 0.3 µm thick layer of ZnO was sputtered on top of the bottom electrode and patterned into ring shapes with inner and outer diameters of 30 µm and 80 µm respectively by wet etching (Figure 3d). The top Cr/Au electrode layer was deposited by e-beam evaporation and patterned by a lift-off process (Figure 3e,f). Finally, the LTO sacrificial layer was wet etched (Figure 3g). A top view of two adjacent elements of the fabricated PMUT is shown in Figure 3h.

ZnO was used in this process but other thin film piezoelectric materials such as PZT can also be adopted. However, limitations still exist regarding the lateral etching rate of the sacrificial layer and the compatibility of the membrane stack with the etching processes for the sacrificial layers [46]. Therefore, many recent PMUTs have been developed with other processes for diaphragm formation.

### 3.2. Diaphragm Defined with Back-Side Etching

An alternative is to define the diaphragm with back-side etching. The etching step itself can take place either before deposition of the piezoelectric layer [28,30,64,79,80,81] or after completing the whole membrane stack [26,31,52]. Small variations in the design of these two processes are mainly due to the initial substrates and different etch stops. In many early devices, a boron-doped Si layer was used as the etch-stop layer [26,30,57]. Alternatively, silicon on insulator (SOI) wafers can be employed with a tailored thickness of the device layer, with the buried oxide layer then used as an etch stop [28,32,33,69,70,73,75].

**Figure 4:**
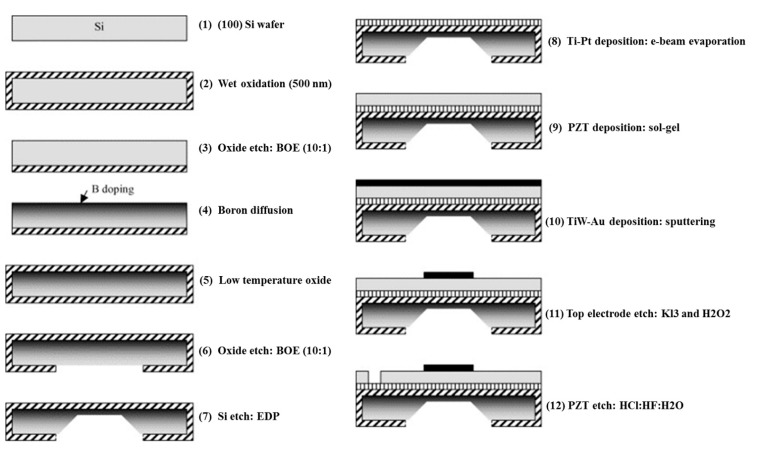
Fabrication process flow of PMUT element with diaphragm defined by back-side etching © 2004 Elsevier B.V. Reprinted with permission from [30].

Figure 4 shows a fabrication process flow starting with a (100) Si wafer [30]. As is the case for surface micromachining, the process begins with preparation of the insulator (e.g., SiO_2_ or Si_3_N_4_) on the silicon. This is then etched from one side of the Si in preparation for B doping. B diffusion occurs at a specific rate, allowing control of the junction depth. After doping, the surface is cleaned then coated with LTO.

Subsequently, standard photolithography is used to pattern the back-side etch window. Later, the wafer is etched with an etchant such as ethylenediamine-pyrocatechol-water-pyrazine (EDP). After the back-side etching, a Ti/Pt bottom electrode is deposited by e-beam evaporation, followed by deposition of PZT and the top electrode. Finally, the top electrode and PZT are etched separately to pattern the top electrode and access the bottom electrode.

In this process, EDP is used as the etchant, but other anisotropic wet etchants can also be used, e.g., potassium hydroxide (KOH) [31,52,82] and tetramethylammonium hydroxide (TMAH) [46,83], depending on the etch stops and the requirement of etch rate selectivity.

**Figure 5:**
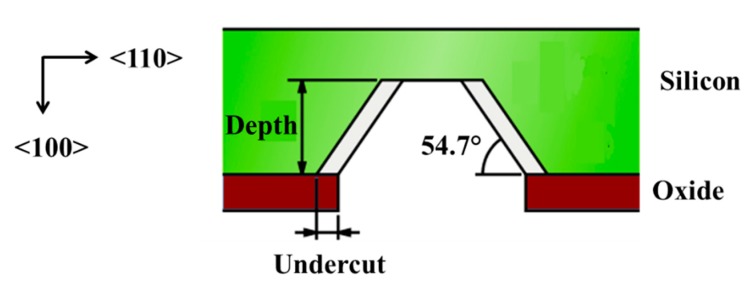
Sloping sidewalls are formed with anisotropic wet etching.

One of the disadvantages of the anisotropic etching in this process is the creation of 54.7° sloping sidewalls [30], as shown in Figure 5. This limits the minimum size of diaphragm and the pitch of the transducer array the process can create. In turn, this constrains the maximum frequency of the transducer array that can be obtained because, ideally, a half-wavelength (λ/2) pitch is required to avoid grating lobe effects in ultrasound imaging applications. Therefore, the back-side etching in current devices is usually performed with deep reactive-ion etching (DRIE), obtaining relatively perpendicular side walls [32,33,35,59,60,64,70], and this works well with SOI wafers using the buried oxide as the etch stop [32,33,70].

### 3.3. Diaphragm Defined with Front-Side Etching

**Figure 6:**
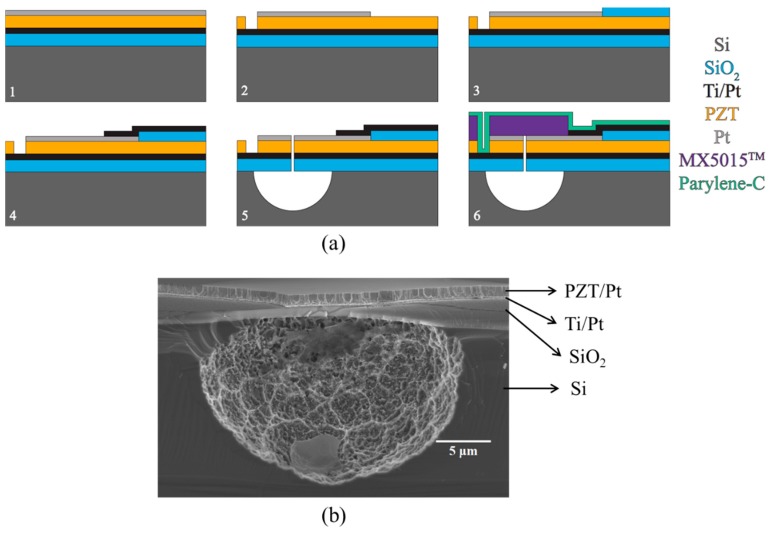
(**a**) Fabrication process flow of PMUTs with diaphragm defined by front-side etching; (**b**) FESEM cross-sectional image of a released cavity.

An alternative route to define the diaphragm cavity is to etch the Si substrate through the membrane from the front (surface micromachining) [36,67]. Figure 6 shows a fabrication process flow for PMUTs with circular diaphragms released from the front-side. Here, the underlying Si substrate was isotropically etched through predefined etch vias. The final membrane of the fabricated PMUTs comprised a SiO_2_/Ti/Pt/PZT/Pt polymer stack.

A 1 μm thick layer of PZT was grown on a commercial platinized Si wafer by RF sputter deposition at room temperature. The wafer had 1 μm thick thermal SiO_2_, 20 nm of Ti and 150 nm of (111) oriented Pt, and was thermally cleaned prior to the deposition. To avoid film cracking and improve density, the film was grown in three successive layers of thicknesses 500 nm, 250 nm and 250 nm, annealed in O_2_ after each growth, as seen in Figure 2. Then a layer of Pt was sputtered on the PZT as the top electrode and annealed at 500 °C to improve adhesion. The top electrode was patterned via contact lithography and RIE. The access to the bottom Ti/Pt electrode was achieved by reactive ion etching (RIE) of the exposed PZT using a thick photoresist mask.

Later, before the fabrication of the electrode fan-out, a layer of SiO_2_ was prepared as an insulation pad on the exposed PZT by sputter deposition and lift-off, reducing potential parasitic capacitance associated with the electrode fan-out and bond pads. Ti/Pt was then sputtered and patterned via lift-off to create a conformal connection to the top electrode of the PZT. Etch vias were created to access the bare silicon beneath the stack to allow for front-side release of the diaphragms. The stack of Pt, PZT, Ti/Pt and SiO_2_ was etched with RIE.

To allow multiple diameters of diaphragms and to avoid damaging the fragile membranes after release, the wafer was diced prior to further steps. Immediately prior to release, any native oxide on the bare Si was removed with a brief CF_4_ etch to ensure symmetrical cavities. The diaphragms were then defined with XeF_2_ isotropic etching. Depending on the number of XeF_2_ etching cycles, different diameters can be obtained. A Ø35 μm hemispheric cavity is shown in Figure 6b.

To prevent backfill of the etch cavity and improve acoustic performance, the diaphragms were laminated with a 15 μm thick negative photoresist film (MX5015, DuPont Electronic Technologies, Hertfordshire, UK) to seal the vias. Prior to lamination, the dies were cleaned with an O_2_ plasma to improve laminate adhesion. The laminate was then patterned to expose the bond pads for wire bonding. After wire bonding, the complete packages were cleaned with an Ar plasma then coated with parylene. To improve the hydrophilicity of the parylene, the surface was activated in an O_2_ plasma for several seconds.

The advantage of this front-side release process is that cavities of relatively smaller diameter can be achieved easily. As mentioned earlier, the necessary element pitch of high frequency transducer arrays imposes limits on the size of PMUT cavities, e.g., diameter <50 μm for frequency >30 MHz. However, an additional layer must be added to the device after diaphragm fabrication to seal the etch vias through the membrane. This layer in turn increases the effective stiffness of the membrane, increasing the resonant frequency of the PMUTs.

### 3.4. Wafer Transfer Diaphragm Formation

Another approach to diaphragm formation is to fabricate the cavity and the PMUT membrane independently on two substrates and bond the two parts together [71,79,84,85].

**Figure 7:**
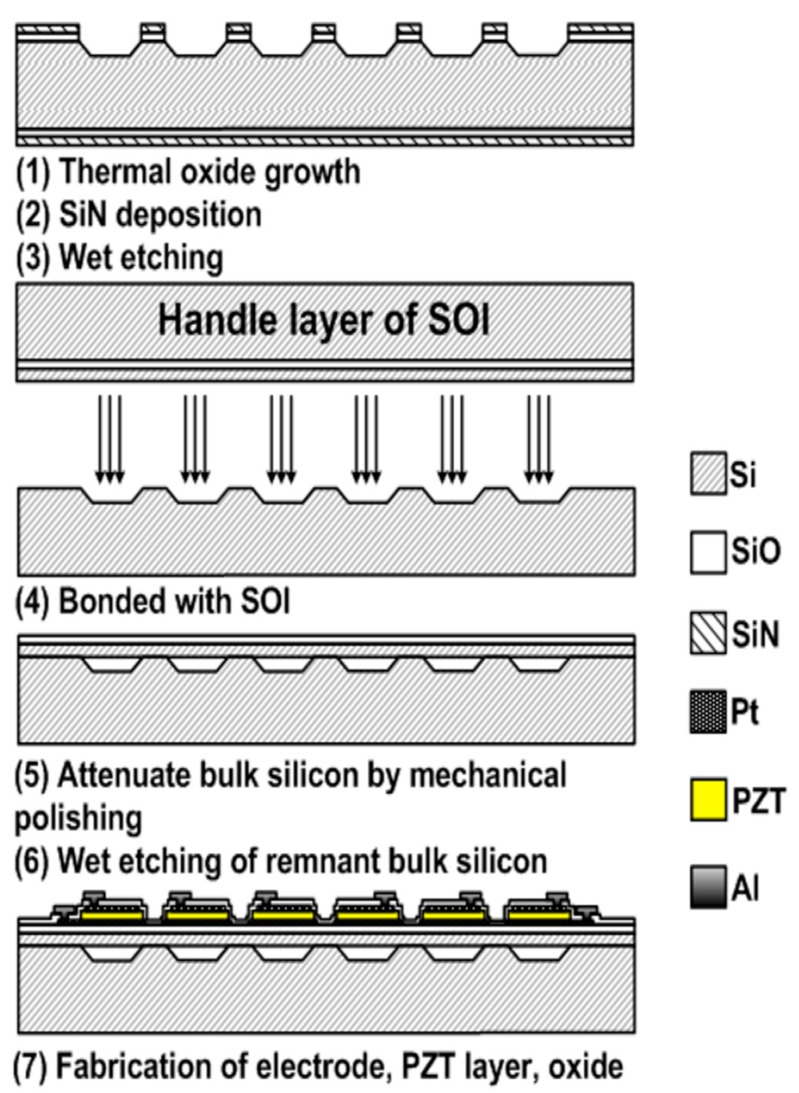
Fabrication process flow of PMUTs with diaphragms formed by reverse bonding [71].

Figure 7 shows the fabrication process flow for PMUTs with diaphragms formed by such a bonding process [71]. First, SiO_2_ and Si_3_N_4_ are grown on a Si substrate. After patterning, high-density cavities and air channel structures are etched on one side of the substrate, e.g., using KOH.

The air channel structures are used for compensation of the air pressure in the sealed cavities, preventing potential damage of the membrane stack during annealing. Subsequently, a SOI wafer is bonded on top of the Si substrate with the cavities at atmospheric pressure. The device layer of SOI is left as the supporting structure for the cavities. Then the handle layer of SOI is removed by chemical mechanical polishing (CMP) and wet etching processes to expose the buried oxide layer.

**Figure 8:**
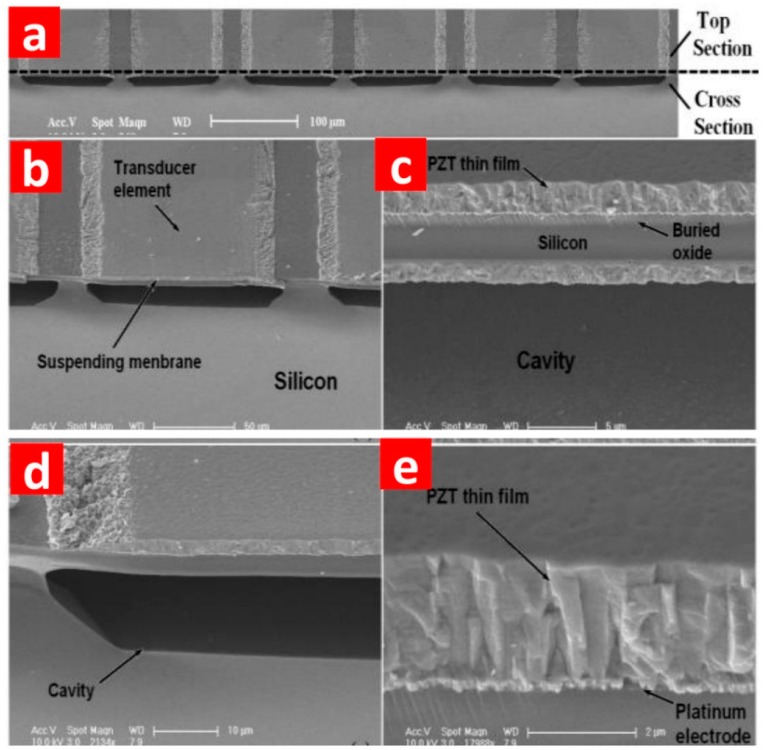
SEM images of (**a**) oblique view of the structure of PMUTs; (**b**) oblique view of a single PMUT element; (**c**) the suspended membrane stack; (**d**) oblique view of the cavity; and (**e**) the PZT thin film layer [71].

The bottom electrode (Ti/Pt), PZT and top electrode layers are deposited and patterned subsequently on the buried oxide layer with the Ti/Pt electrode also acting as a barrier layer to prevent inter-diffusion between PZT and SiO_2_. Then a thin layer of SiO_2_ is deposited by PECVD to protect the top electrode and PZT during the fabrication of the Al electrode fan-out and bond pads. This completes the PMUTs with a membrane stack of Si/SiO_2_/Ti/Pt/PZT/Pt suspended above the cavities as shown in Figure 8.

In this process, the practical size of the PMUT element is defined by the initial cavity etching. Thus the main constraint on the interspace between two adjacent PMUT elements is the accuracy of lithography and the alignment through the whole process [71,79,84,85].

## 4. Development of PMUT Arrays

Generally, a transducer with a single element cannot fully satisfy many practical applications, especially in medical imaging and NDE. Instead, the use of multiple transducer elements and dynamic control of each element is used to achieve electronic beam steering, focusing and scanning, which bring benefits in reduction of test time and better reliability and quality of measurements.

**Figure 9:**
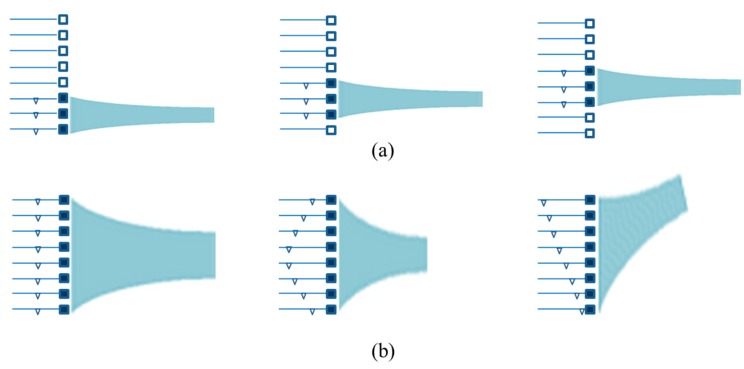
Schematic diagrams of (**a**) electronic scanning of 1-D linear arrays; **(b**) electronic focusing and steering of 1-D phased linear arrays.

Linear arrays, Figure 9, have a number of transducer elements arranged in a line. Each element usually has the same geometrical shape with width in the lateral direction and height in the elevation direction. The distance between the centers of two adjacent elements is defined as the pitch, and the gap between them is the kerf. The pitch is often smaller than λ, ideally λ/2, to avoid grating lobe effects which will degrade the image quality. In the operation of a linear array, only a subset of elements is activated each time to transmit or receive, and a scan is realized by altering the selection of active elements, as shown in Figure 9a. The ultrasound beam is transmitted perpendicular to the element surface, giving a smaller activated aperture than the whole transducer. If a phase delay is introduced into the signal applied to each element of the linear array, ultrasound beam focusing and steering can be achieved, as shown in Figure 9b. Linear arrays with phase delay control in which all the elements of the array are active as one set are called phased linear arrays.

**Figure 10:**
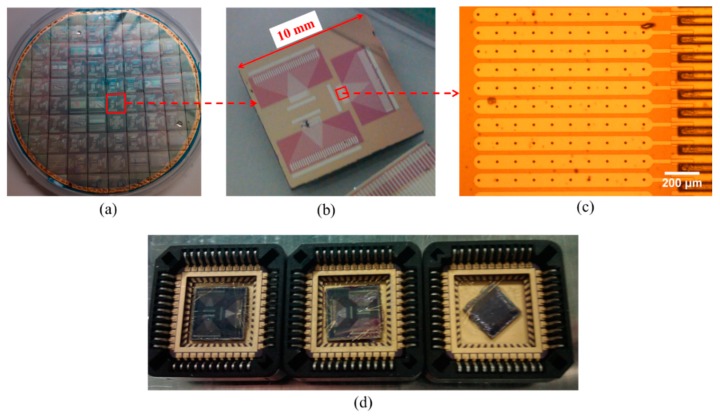
Images of (**a**) a 4-inch wafer with multiple PMUT dies with different diameter diaphragms; (**b**) three PMUT arrays on one die; (**c**) several elements of a PMUT array, each consisting of ten diaphragms; and (**d**) three fully packaged wire-bonded devices.

For 1-D linear PMUT arrays, each array element is usually formed by connecting multiple PMUT elements in parallel and driving them together. Figure 10 shows 32-element PMUT arrays fabricated by the authors for high frequency applications through the fabrication process detailed in Section 3.3. Each PMUT array element consisted of ten Ø80 µm PMUT elements with shared electrodes.

To test the 32-element PMUT arrays, a conventional un-matched PZT piezocomposite transducer of 30.5 MHz center frequency and 36% −6 dB fractional bandwidth [86] was used, first with the PMUT operating as a receiver, with the piezocomposite transducer transmitting, then with the PMUT operating as a transmitter, with the piezocomposite receiving. Because the −6 dB ultrasound beam diameter of the piezocomposite transducer was about 80 µm at the focal point, thus the beam was incident principally only on one or two diaphragms in the PMUT array element.

**Figure 11:**
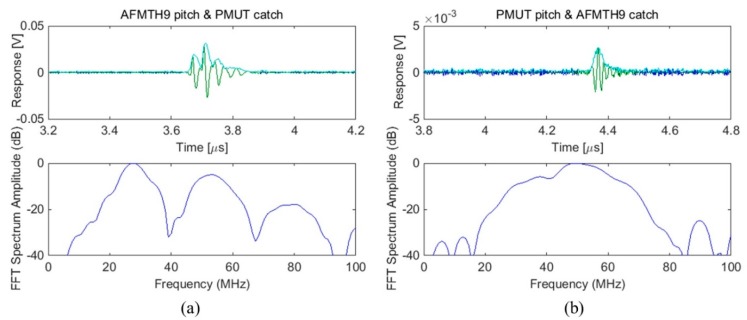
(**a**) Receive and (**b**) transmit response of one element in a PMUT array measured with a 30.5 MHz piezocomposite transducer in deionized water. In the time-domain figures, the blue curve represents original signal with green part of Tukey-windowed signal, and the cyan curve represents Hilbert transformed signal; in the frequency-domain figures, the blue curve represents the FFT spectrum of the Tukey-windowed signal.

Therefore, a five degree-of-freedom platform was used to fine tune the alignment of the transmitter and the receiver, *i.e*., orientation and position, in order to achieve better SNR and to maximize the signal amplitude of the received pulse. The distance between the transmitter and the receiver was close to the focal length of the piezocomposite transducer, ~4 mm. The acoustic response of the PMUT array shown in Figure 11, on both reception and transmission, demonstrates the achievement of reasonable sensitivity and relatively large bandwidth.

**Figure 12:**
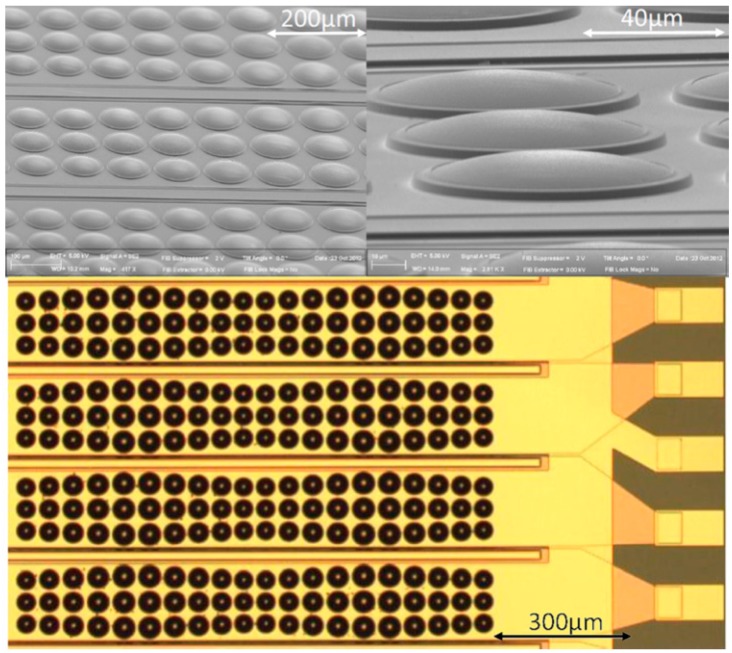
SEM and optical images of a 64 channel 5 MHz linear array exploiting five different dome sizes with cavities ranging from 74 to 90 µm diameter. © 2012 AIP Publishing LLC. Reprinted with permission from [34].

It was noticed that the center frequency and −6 dB bandwidths in reception were close to the pulse-echo response of the piezocomposite transducer, but smaller than those in the transmit response. This result was anticipated, because the frequency and bandwidth of the Ø80 µm PMUT diaphragms were expected to be greater than those of the un-matched piezocomposite transducer.For lower frequency PMUT arrays, multiple lines of PMUT elements can be integrated into one array element [34], as shown in Figure 12. Moreover, by arranging pre-shaped PMUTs with slightly different diameters, a broader bandwidth and improved sensitivity can be realized through the complex interaction between the individual PMUT elements [34,65].

**Figure 13:**
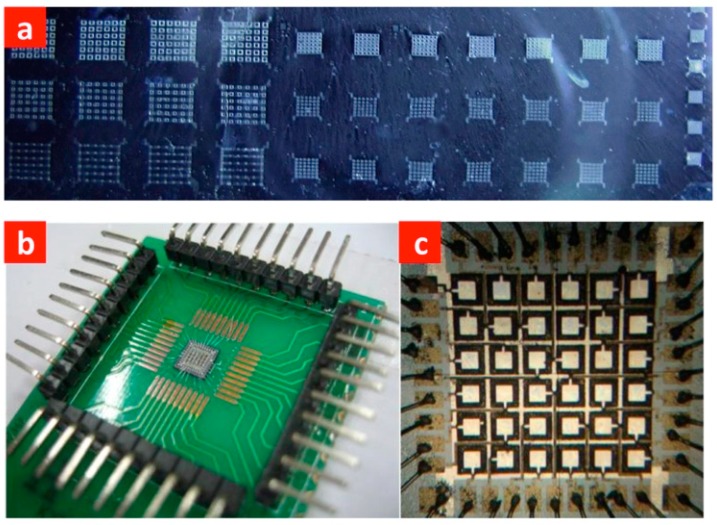
Photographs of (**a**) 2D PMUT arrays; (**b**) a packaged array used for testing; and (**c**) a 6 × 6 2-D PMUT matrix array with element size 200 μm × 200 μm and total area less than 2 mm × 2 mm [71].

The principle of 2-D arrays is similar to that of linear arrays, but with multiple elements in the elevation direction so that the beam can be controlled dynamically in two orthogonal directions with appropriate phased delays. Therefore, 2-D arrays usually have a large number of elements forming a matrix. The ultrasound beam can be fully steered in 2-D, allowing maximum electronic control flexibility. However, the electrical interconnection is more difficult than 1-D arrays, as individual electrical addressing is usually required for each element, as shown in Figure 13 [71]. An alternative is to use a crossed-electrode array configuration with row-column addressing [70] to reduce the complexity of interconnects, but control flexibility is also constrained, reducing array performance.

**Figure 14:**
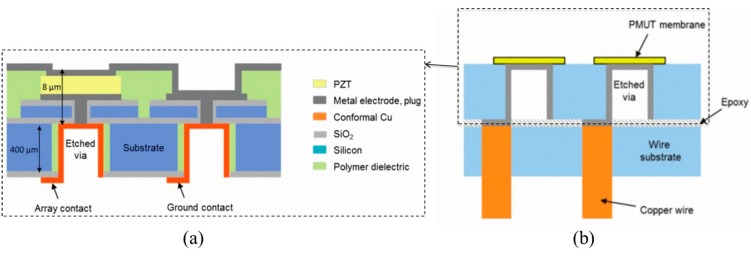

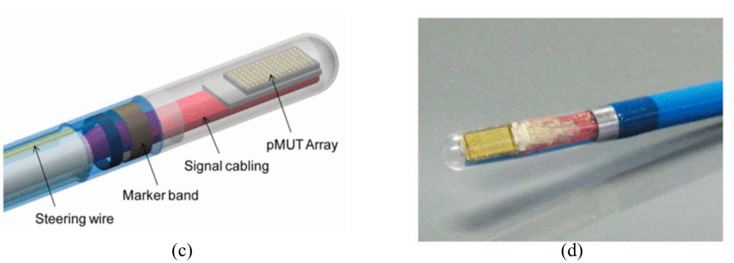
Cross-sectional schematic diagrams of (**a**) PMUTs with through-Si interconnects and (**b**) a PMUT array substrate bonded onto a wiring substrate; (**c**) a mechanical model and (**d**) photograph of the distal end of a steerable 14-Fr (Ø4.667 mm) ICE catheter containing a 512-element PMUT matrix array. © 2013 IEEE. Reprinted with permission from [35].

More recently, Dausch *et al.* [35] have successfully developed intracardiac echocardiography (ICE) catheters integrated with 5 MHz PMUT matrix arrays of 256 and 512 elements, as shown in Figure 14. Through-Si interconnects were adopted to allow individual addressing of each element in a constrained space. This allowed demonstration of real-time *in vivo* 3-D imaging using a PMUT matrix array integrated device in which the electrodes of the PMUT elements were led to the back surface of the PMUT substrate a), and thermo-compression bonding was used to bond the PMUT substrate onto the wiring substrate with epoxy (Figure 14b). This interconnection scheme not only enables good electrical interconnection to the PMUTs without significantly increasing electronic footprint or using other time-consuming interconnect means (e.g., wire-bonding), but also further reduces the overall dimensions of PMUT array devices [35].

## 6. Conclusions and Outlook

In recent years, increasing attention has been given to MUTs because of their advantages over conventional ultrasound transducers, such as miniaturization, low *Z_ac_*, high bandwidth and sensitivity, and high electromechanical coupling coefficient, and with potential for automated large batch production and integration with front-end electronics. Both CMUTs and PMUTs have been explored; the latter require deposition of piezoelectric layers but are less limited by geometric and electronic constraints, with reduced power consumption.

Compared to the conventional ultrasound transducers and CMUTs, only a relatively limited number of publications on PMUTs has appeared over the last 25 years, which may be attributed to two reasons: the difficulties in the manufacture of high performance piezoelectric thin films, and the lack of efficient modelling methods or tools for accurate prediction of the effects of intrinsic stress. These challenges have therefore led to developed PMUTs with lower performance in practice than theory. However, substantial progress has been made to improve the performance, with a particular focus on innovative fabrication approaches [34,35,65], device structures [32,34,60,65,70,73] and drive methods [33,34], and better models [34,53,55].

Continuing research in PMUTs will focus on further improvement of interconnect solutions and acoustic performance (e.g., sensitivity and bandwidth), matching the development of other miniaturized devices to address unmet needs in industry and medicine, e.g., in ultrasound capsule endoscopy [87].
